# Supplementary material for: Temporal and spatial variability of terrestrial diatoms at the catchment scale: controls on productivity and comparison with other soil algae
Source: PeerJ. 2020 Jun 8;8:e9198. doi: 10.7717/peerj.9198 (PMC7289147; doi:10.7717/peerj.9198)
Supplement: Supplemental Information 3 — Land use, soil type and average pH values are given for the 16 sites. [file peerj-08-9198-s003.docx]

| **Site** | **Habitat** | **Soil type** | **pH** |
| --- | --- | --- | --- |
| 1 | agricultural grassland | sandstone | 7.66 |
| 2 | forested | sandstone | 5.73 |
| 3 | undisturbed grassland | marls | 6.32 |
| 4 | forested | schists | 6.37 |
| 5 | agricultural field | schists | 6.32 |
| 6 | agricultural field | marls | 7.63 |
| 7 | forested | schists | 5.17 |
| 8 | agricultural grassland | schists | 6.53 |
| 9 | agricultural grassland - agricultural field | marls | 6.92 |
| 10 | agricultural grassland | Luxembourg red sandstone | 6.77 |
| 11 | agricultural grassland -  agricultural field | schists | 6.30 |
| 12 | forested | marls | 7.01 |
| 13 | undisturbed grassland -  grazed grassland | alluvial | 5.97 |
| 14 | forested | alluvial | 7.59 |
| 15 | grazed grassland | marls | 6.60 |
| 16 | agricultural field | schists | 5.81 |
